# PfHRP2 Measures Schizogony, Not Mechanical Blockage

**DOI:** 10.1371/journal.pmed.0030068

**Published:** 2006-01-31

**Authors:** Ian Clark

**Affiliations:** **1**Australian National UniversityCanberra, Australian Capital TerritoryAustralia

## Abstract

n/a

As noted in Dondorp et al. [ 1], histidine-rich protein 2 (PfHRP2) is released at schizont rupture as part of the regular 48-hour developmental cycle of the erythrocytic form of the parasite. Since this release of PfHRP2 into the circulation occurs while the parasitized red cell is adhering to vascular endothelium, it can act as an indirect marker for this sequestration. Therefore, as might be expected for a parasite that sequesters for a fixed part of its repeated 48-hour cycle of development, both the total biomass and sequestered biomass were calculated to be associated with severity of disease.

The authors use these data to further the case for the traditional concept that disease symptoms in falciparum malaria—including coma, high lactate, and renal failure—arise because erythrocytes containing mature forms of the parasites sequester within the microvasculature of the vital organs. We may safely infer from the authors' previous publications their acceptance of the conventional wisdom that this sequestration mechanically obstructs vessels, leading to tissue hypoxia through poor oxygen transport.

Parasites are inside sequestering red cells when they burst and release PfHRP2, but it may be bursting, not sequestration, which matters most in disease pathogenesis. PfHRP2 is a marker for the degree of schizogony not, as implied, of vascular blockage caused by sequestration. Clinical tolerance to falciparum malaria, common in endemic areas in age groups with high parasite densities, demonstrates this well. Those who champion mechanical vascular obstruction must accept this as a state in which appreciable sequestration occurs only in harmless locations, such as larger veins and nonvital organs. It is not known where red cells containing mature parasites lodge in these individuals, and if they stop using these locations during serious illness. If they do not stop, PfHRP2 released from schizonts adhering in harmless locations would add to the total concentration in the circulation, but would not be a marker for obstruction.

Other molecules released at schizogony include the trigger(s) that generate the inflammatory cytokines, which have formed the basis of a mainstream argument for the pathophysiology of malarial disease for the past 25 years (see [ 2, 3, 4] for recent reviews). An undiscussed reason for PfHRP2 release correlating with serious illness might be its value as a surrogate for these cytokine-triggering molecules liberated from bursting red cells postschizogony. An awareness of these concepts has allowed molecules of host origin, such as increased plasma levels of the soluble form of one of the receptors for tumor necrosis factor, to be considered alongside PfHRP2 as a marker for the parasite biomass [ 5].

If the cultural gap between the mechanical and the cytokine approach to malarial disease could be spanned, useful knowledge on roles of inflammatory cytokines in sepsis, such as details of how cytokine-induced mitochondrial dysfunction causes a functional hypoxia [ 6, 7], could more readily be applied to understanding malarial disease.

## References

[pmed-0030068-b1] Dondorp AM, Desakorn V, Pongtavornpinyo W, Sahassananda D, Silamut K (2005). Estimation of the total parasite biomass in acute falciparum malaria from plasma PfHRP2. PLoS Med.

[pmed-0030068-b2] Clark IA, Cowden WB (2003). The pathophysiology of falciparum malaria. Pharmacol Ther.

[pmed-0030068-b3] Maitland K, Marsh K (2004). Pathophysiology of severe malaria in children. Acta Trop.

[pmed-0030068-b4] Boutlis CS, Riley EM, Anstey NM, de Souza JB (2005). GPI in malaria pathogenesis and immunity: Potential for therapeutic inhibition and vaccination. Curr Topics Microbiol Immunol.

[pmed-0030068-b5] Ochola LB, Marsh K, Lowe B, Gal S, Pluschke G, Smith T (2005). Estimation of the sequestered parasite load in severe malaria patients using both host and parasite markers. Parasitology.

[pmed-0030068-b6] Singer M, De Santis V, Vitale D, Jeffcoate W (2004). Multiorgan failure is an adaptive, endocrine-mediated, metabolic response to overwhelming systemic inflammation. Lancet.

[pmed-0030068-b7] Callahan LA, Supinski GS (2005). Sepsis induces diaphragm electron transport chain dysfunction and protein depletion. Am J Resp Crit Care Med.

